# Crystal structure and Hirshfeld surface analysis of (*E*)-2-[(anthracen-9-yl­methyl­idene)amino]-4-nitro­aniline

**DOI:** 10.1107/S2056989026001027

**Published:** 2026-02-05

**Authors:** Soukaina Benkirane, Houria Misbahi, Joel T. Mague, Tuncer Hökelek, Mazzah Ahmed, Nada Kheira Sebbar

**Affiliations:** a Laboratory of Applied Organic Chemistry, Sidi Mohamed Ben Abdellah University, Faculty Of Science And Technology, Road Immouzer, BP 2202 Fez, Morocco; b Department of Chemistry, Tulane University, New Orleans, LA 70118, USA; c Department of Physics, Hacettepe University, 06800 Beytepe, Ankara, Türkiye; d University of Lille, CNRS, UAR 3290, MSAP, Miniaturization for Synthesis, Analysis and Proteomics, 59000 Lille, France; e Laboratory of Heterocyclic Organic Chemistry, Pharmacochemistry Competence Center, Av. Ibn Battouta, BP 1014, Faculty of Sciences, Mohammed V University, in Rabat, Morocco; University of Massachusetts Dartmouth, USA

**Keywords:** crystal structure, π-stacking, C—H⋯π(ring) inter­action

## Abstract

The title compound, C_21_H_15_N_3_O_2_, contains a nitro­aniline ring and an anthracene ring system bridged over the methyl­ene amino group. The anthracene ring system is essentially planar with an r.m.s. deviation of 0.03 (2) Å and it is oriented at a dihedral angle of 79.70 (5)° with respect to nitro­aniline ring. There is an intra­molecular N—H⋯N hydrogen bond between N atoms of nitro­aniline ring and amino group. In the crystal, N—H—O hydrogen bonds link the mol­ecules into infinite chains along the *b*-axis direction. π–π stacking inter­actions between the nitro­aniline rings of adjacent mol­ecules with centroid-to-centroid distance of 3.7682 (2) Å and C—H⋯π(ring) inter­actions may help to consolidate the three-dimensional architecture. A Hirshfeld surface analysis indicates that the most important contributions for the crystal packing are from H⋯H (35.5%), H⋯C/C⋯H (33.7%) and H⋯O/O⋯H (18.3%) inter­actions.

## Abstract 2

The title compound contains a nitro­aniline ring and an anthracene ring system bridged over the methyl­ene amino group. In the crystal, the N—H⋯O hydrogen bonds link the mol­ecules into infinite chains along the *b*-axis direction. π–π stacking inter­actions between the nitro­aniline rings of adjacent mol­ecules with centroid-to-centroid distance of 3.7682 (2) Å and C—H⋯π(ring) inter­actions are also observed.

## 1. Chemical context

The Schiff base family is a class of organic compounds characterized by the presence of an imine group (Moss *et al.*, 1995 ▸; Schiff, 1864 ▸). Their structural features confer notable reactivity and versatility, making them valuable scaffolds with numerous applications, such as fluorescent chemosensors (Udhayakumari *et al.*, 2020 ▸), as catalysts (Boghaei *et al.*, 2002 ▸), in water treatment (Khan *et al.*, 2019 ▸) and as corrosion inhibitors (Ashassi-Sorkhabi *et al.*, 2005 ▸; Verma & Quraishi, 2021 ▸). In medicinal chemistry, numerous investigations have also highlighted their broad spectrum of activities (Hameed *et al.*, 2017 ▸; Mushtaq *et al.*, 2024 ▸; Nidhi *et al.*, 2025 ▸; Younus *et al.*, 2023 ▸), notably as anti-microbial (Barakat *et al.*, 2025 ▸), anti­cancer (Uddin *et al.*, 2020 ▸), anti-inflammatory (Murtaza *et al.*, 2017 ▸), anti­viral (Azzouzi *et al.*, 2024 ▸), anti-diabetic (Adalat *et al.*, 2022 ▸), and anti­oxidant (Madi *et al.*, 2021 ▸) agents. Particular focus on derivatives incorporating nitro­benzene or anthracene moieties has demonstrated significant activities (Aravindan *et al.*, 2021 ▸; Bai *et al.*, 2017 ▸; Gümüş *et al.*, 2020 ▸; Kraicheva *et al.*, 2012 ▸; Mahmoud *et al.*, 2018 ▸). Prompted by these findings, the title compound was synthesized by the condensation of anthracene-9-carbaldehyde and 4-nitro­benzene-1,2-di­amine, giving a new Schiff compound containing both anthracene and nitro­benzene moieties. Its synthesis and mol­ecular and crystal structures are described here, along with the results of a Hirshfeld surface analysis.
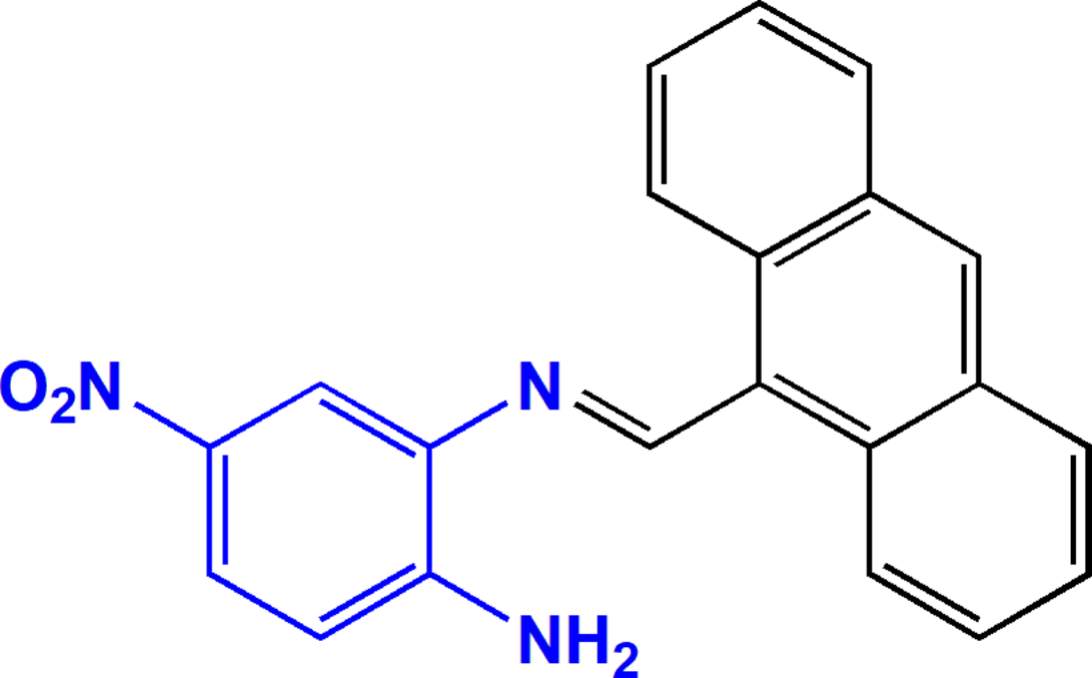


## 2. Structural commentary

**Figure 1:**
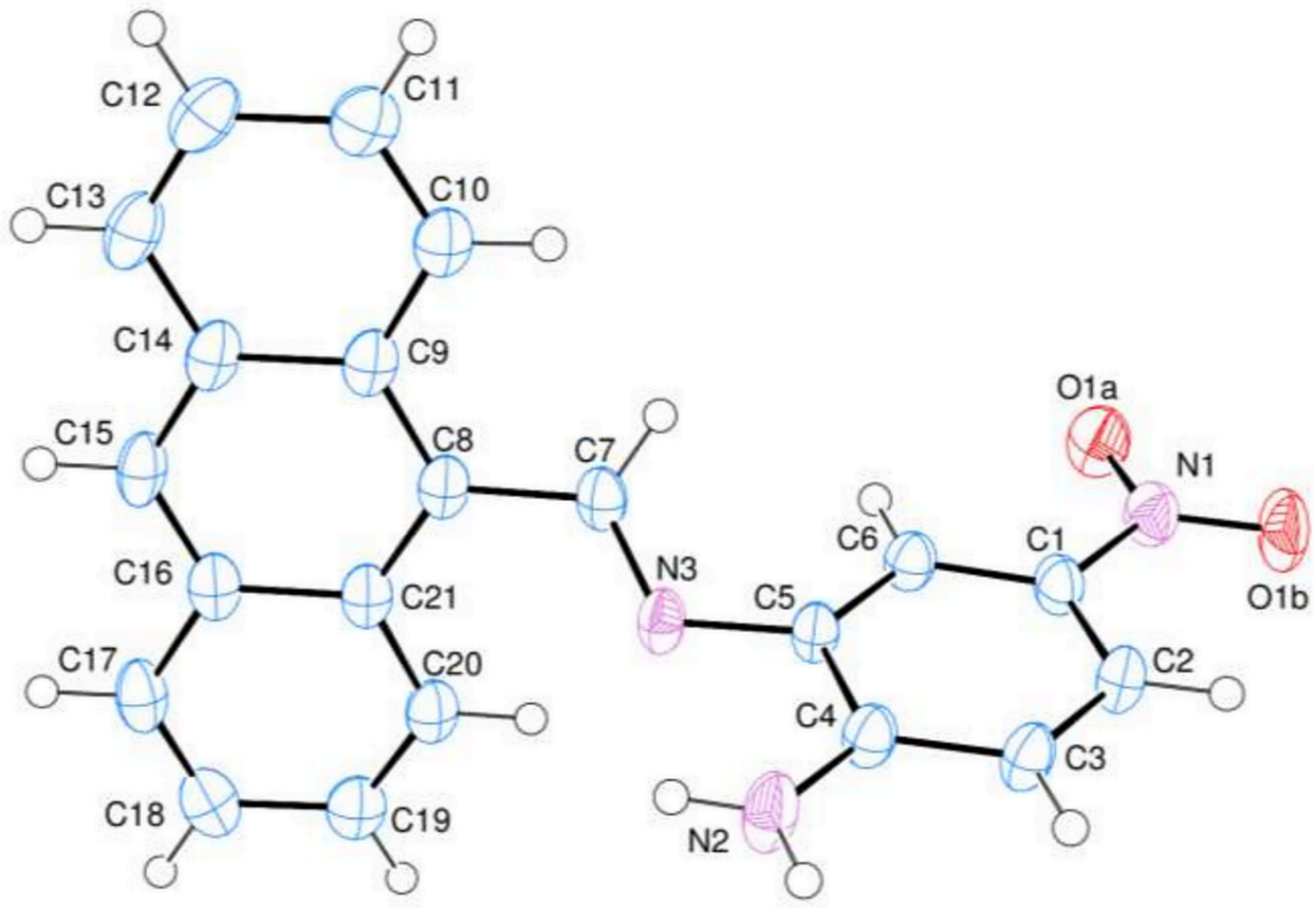
The mol­ecular structure of the title compound (I) showing 50% probability ellipsoids.

The title compound, (I), contains an nitro­aniline ring (*A*, C1–C6) and an anthracene ring system (*B*, C8–C21) bridged over the methyl­ene amino group (Fig. 1 ▸). The anthracene ring system, consisting of three fused benzene rings, is essentially planar with r.m.s. deviation of 0.03 (2) Å and it is oriented at a dihedral angle of 79.70 (5)° with respect to nitro­aniline ring *A*. Atoms N1, N2, N3, O1*A* and O1*B* are 0.0345 (18), 0.0135 (20), 0.0168 (16), 0.1040 (17) and −0.0276 (18) Å, respectively, away from the best least-squares plane through ring *A*. Thus, they are nearly coplanar. There is an intra­molecular N–H⋯N hydrogen bond (Table 1 ▸) between the N atoms of ring *A* and the amino group. No unusual bond lengths or inter­bond angles are observed.

## 3. Supra­molecular features

**Figure 2:**
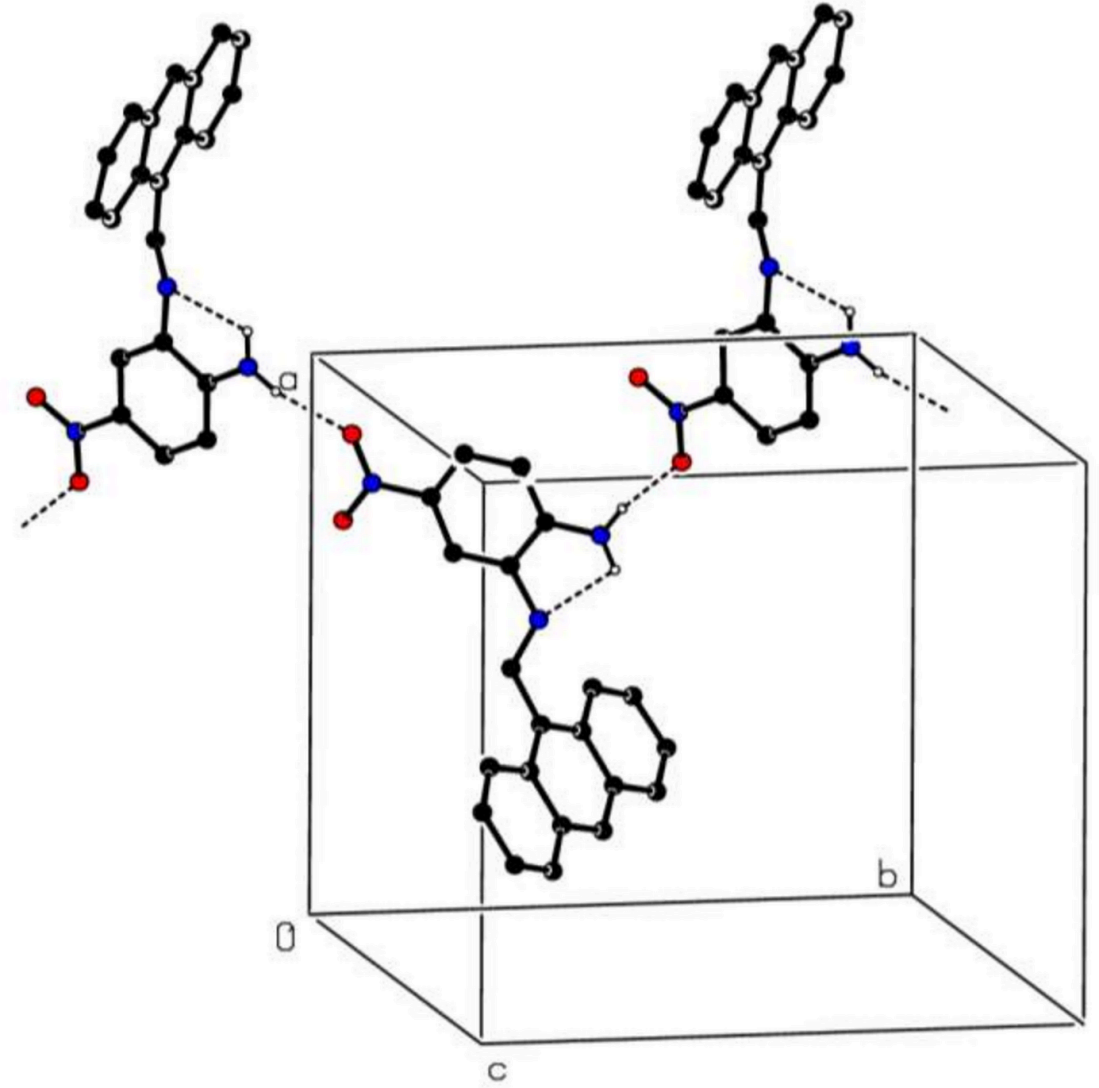
A partial packing diagram of the title compound (I). The intra­molecular N—H⋯N and inter­molecular N—H⋯O hydrogen bonds are shown as dashed lines. Hydrogen atoms not involved in these inter­actions have been omitted for clarity.

In the crystal, N—H—O hydrogen bonds (Table 1 ▸) link the mol­ecules into infinite chains along the *b*-axis direction (Fig. 2 ▸). π–π stacking inter­actions between the *A* rings [centroid-to-centroid distance = 3.7682 (2) Å, α = 0.02 (10)° and slippage = 1.375 Å] of adjacent mol­ecules and C—H⋯π(ring) inter­actions (Table 1 ▸) may help to consolidate the three-dimensional architecture.

## 4. Hirshfeld surface analysis

**Figure 3:**
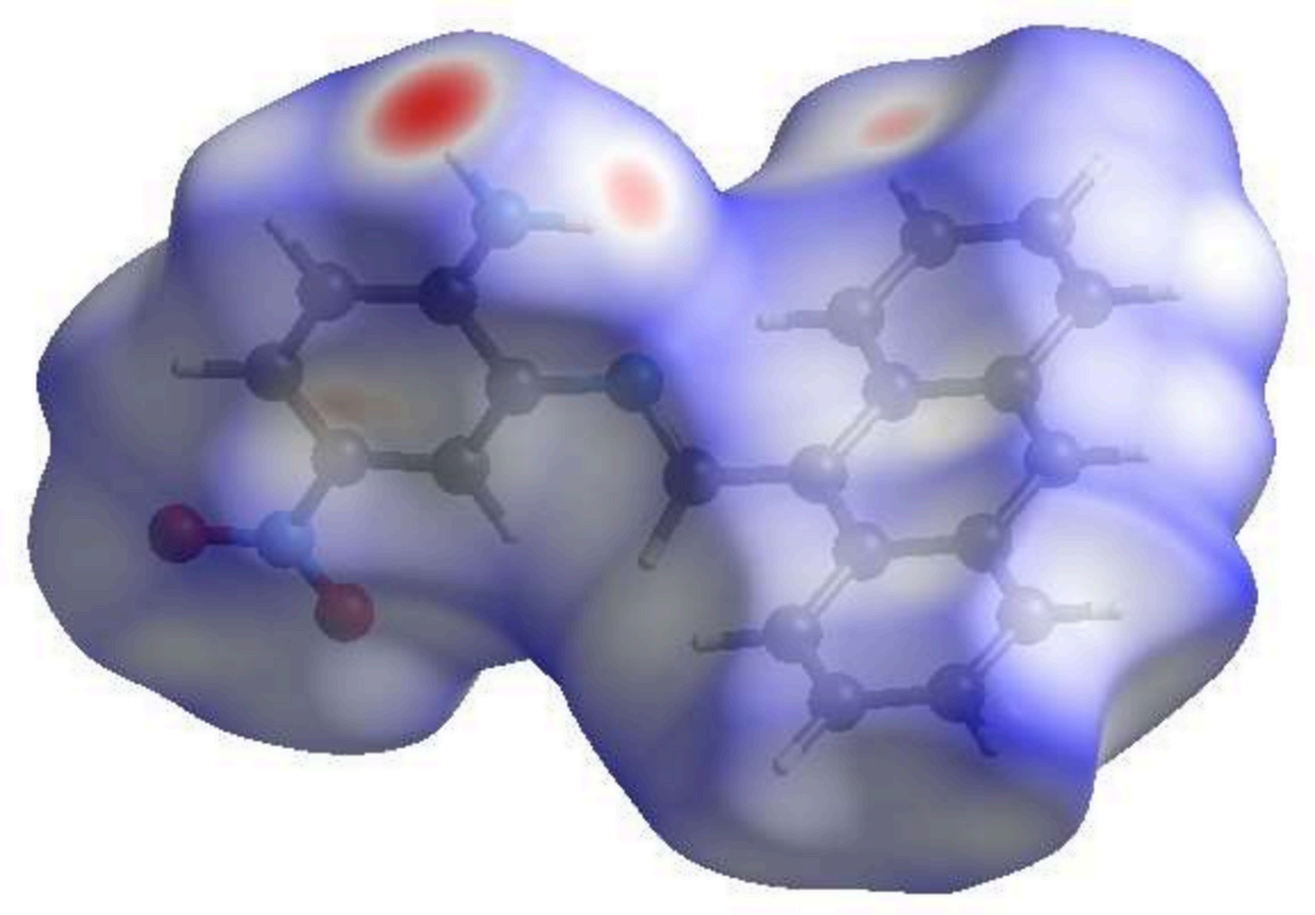
View of the three-dimensional Hirshfeld surface of the title compound (I) plotted over *d*_norm_.

**Figure 4:**
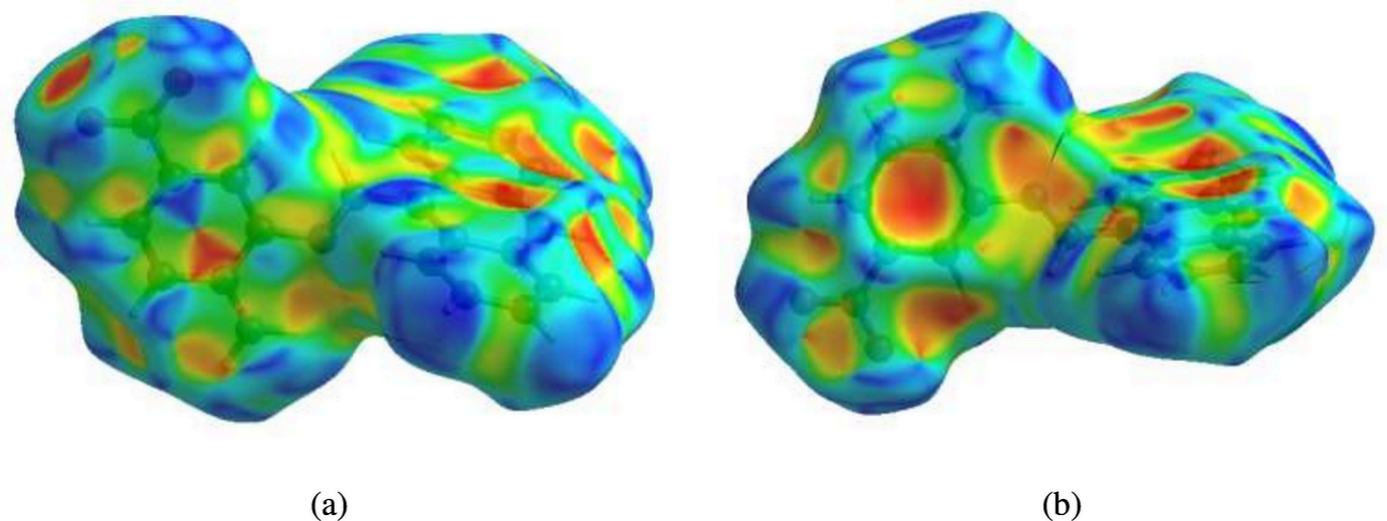
The shape-index surface showing two orientations for (*a*) π–π stacking and (*b*) C—H⋯π(ring) inter­actions.

**Figure 5:**
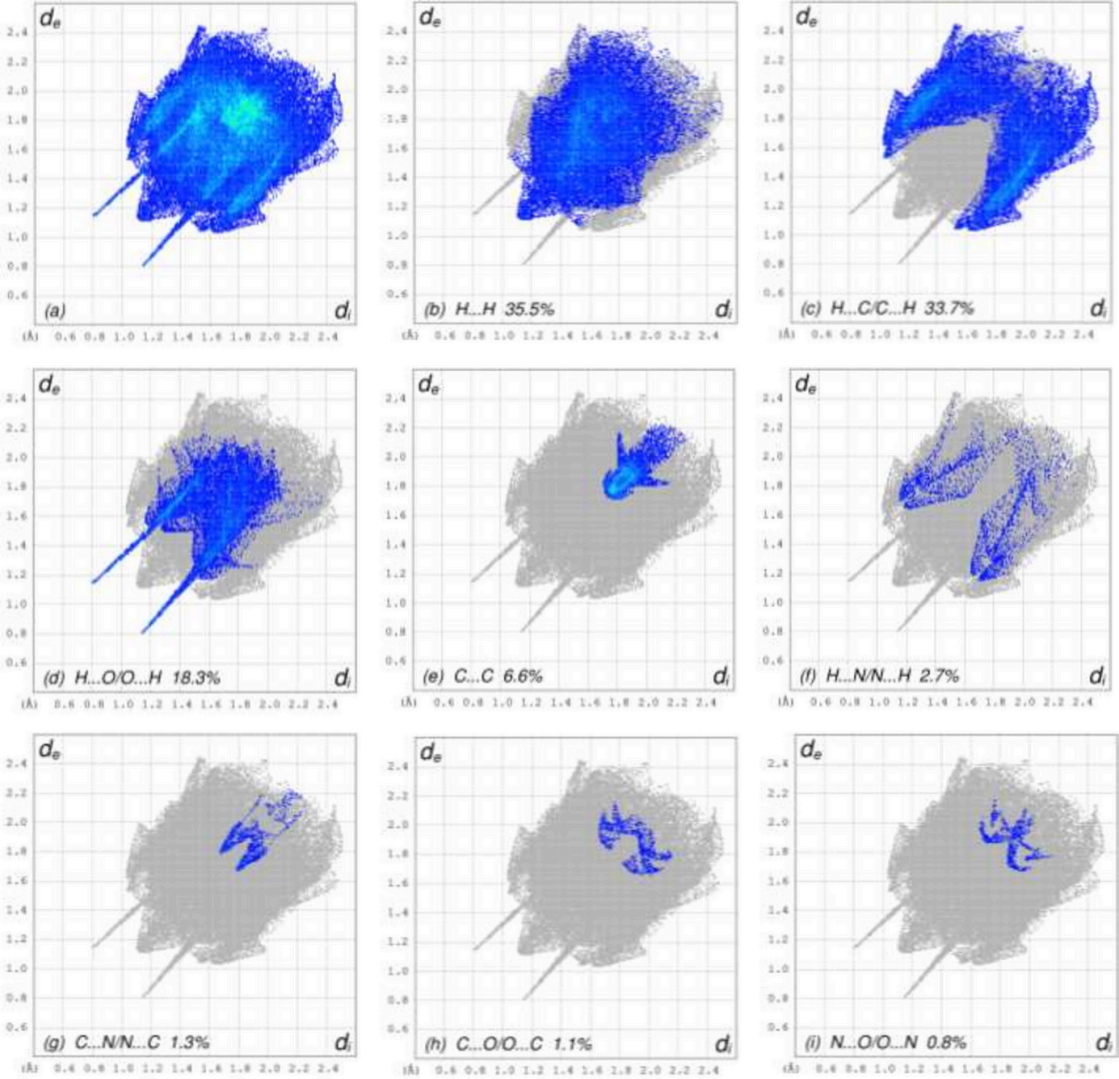
The two-dimensional fingerprint plots of the title compound (I), showing (*a*) all inter­actions, and delineated into (*b*) H⋯H, (*c*) H⋯C/C⋯H, (*d*) H⋯O/O⋯H, (*e*) C⋯C, (*f*) H⋯N/N⋯H, (*g*) C⋯N/N⋯C, (*h*) C⋯O/O⋯C and (*i*) N⋯O/O⋯N inter­actions. The *d*_i_ and *d*_e_ values are the closest inter­nal and external distances (in Å) from given points on the Hirshfeld surface.

A Hirshfeld surface (HS) analysis was carried out using *Crystal Explorer 17.5* (Spackman *et al.*, 2021 ▸) to visualize the inter­molecular inter­actions in the crystal. Fig. 3 ▸ shows the contact distances where the bright-red spots correspond to the respective donors and/or acceptors. The white surfaces and the red and blue areas indicate contacts with distances equal, shorter and longer, respectively, than the van der Waals radii (Table 2 ▸). The π–π stacking and C—H⋯π(ring) inter­actions are shown in Fig. 4 ▸*a* and 4*b* by the presence of the adjacent red and blue triangles and the red π-holes, respectively. According to the two-dimensional fingerprint plots (McKinnon *et al.*, 2007 ▸), the H⋯H, H⋯C/C⋯H and H⋯O/O⋯H contacts make the most significant contributions to the HS, at 35.5%, 33.7% and 18.3%, respectively (Table 2 ▸ and Fig. 5 ▸).

## 5. Database survey

A search of the Cambridge Structural Database (CSD, updated September 2025; Groom *et al.*, 2016 ▸) identified seven compounds with structural similarity to the target compound (*E*)-2-[(anthracen-9-yl­methyl­ene)amino]-4-nitro­aniline. Structures **I** to **VI** (CSD codes: RIRMAH01, LIJQII, WEFBAM, WAZWAX, WAZVUQ and WAZVOK; Geiger & Parsons, 2014 ▸; Goettler & Hamaker, 2022 ▸; Dalapati *et al.*, 2012*a* ▸,*b* ▸) all possess a nitro­benzene ring but do not correspond to Schiff bases. They are distinguished by the nature of their substituents (methyl, phenyl or imidazo[1,2-*a*]pyridin-2-ylmethyl groups) and the possible presence of solvation mol­ecules or anions (H_2_O, H_2_PO_4_^−^, HSO_4_^−^, tetra-*n*-butyl­ammonium). These differences reflect the structural flexibility and the various supra­molecular organizations that the nitro­benzene skeleton can adopt. On the other hand, compound **VII** [CSD refcode: SUYSAH, *N*^2^-(4-chloro­benzyl­idene)-4-nitro­benzene-1,2-di­amine; Farag *et al.*, 2010 ▸] turns out to be the closest structural analogue of the studied compound and shares the same basic Schiff mol­ecular framework formed by the condensation of a 1,2-di­amine derivative and an aromatic aldehyde, as well as a similar electronic arrangement around the azomethine group (–CH=N–). This analysis highlights the structural consistency of the target compound with the analogues listed in the CSD, while highlighting its originality linked to the presence of the anthracene fragment, likely to influence its electronic and π–π stacking properties in the solid state.
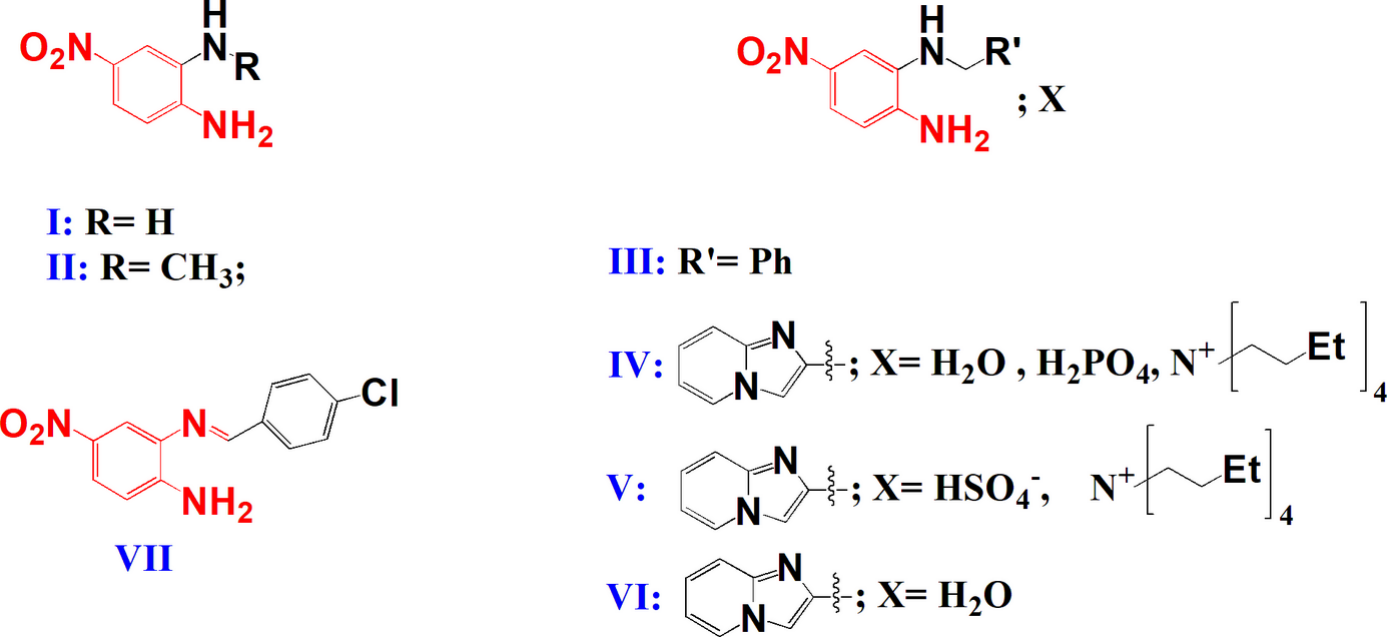


## 6. Synthesis and crystallization

In a flask, 0.4 g (2.61 mmol) of 4-nitro­benzene-1,2-di­amine was stirred into 20 mL of methanol at 323 K until it was completely dissolved. Then, 0.53 g (2.61 mmol) of anthracene-9-carbaldehyde was added in small portions, with the mixture being warmed to reflux. An orange precipitate formed after 1 h, and the reaction was monitored by TLC until the starting materials were consumed entirely (3 h). The mixture was then cooled to ambient temperature, and the precipitate was collected by filtration. It was washed three times (10 mL) with methanol and dried at 323 K to yield a pure powder. The product was characterized by ^1^H and ^13^C NMR, IR and UV-Vis spectroscopy. The slow evaporation of a 2:1 (v/v) mixture of ethyl acetate and methanol was used to obtain single crystals. C_21_H_15_N_3_O_2_**; Colour**: Orange; **Yield**: 98.5%, ***R*****_f_** = 0.72 (ethyl acetate/hexa­ne: 1/1), **Melting Point**: 512.9 K; **^1^H NMR** (DMSO-*d*_6_, 300 MHz): δ (ppm) 6.62 (*s*, 2H, NH_2_), 9.95 (*s*, 1H, –CH=N–), 8.81 (*m*, 3H, H_Ar_), 8.19 (*m*, 3H, H_Ar_), 8.02 (*d*, 1H, ^3^*J*_H–H_*= 9 Hz*, H_Ar_), 7.62 (*m*, 4H, H_Ar_), 6.89 (*d*, 1H, ^3^*J*_H–H_*= 9 Hz*, H_Ar_); **^13^C NMR** (DMSO-*d*_6_, 75 MHz): δ (ppm) 127.62, 130.77, 131.30, 136.72, 151.16 (C_q_), 160.35 (–CH=N–), 113.29, 114.20, 124.93, 125.53, 126.1, 127.97, 129.40, 131.33 (C_Ar_); **FT**-**IR** (cm^−1^): 3450, 3400 (N—H stretching, –NH_2_), 3100, 3000 (aromatic C—H stretching), 1650 (C=N stretching, imine), 1500 (C=C stretching, aromatic ring); **UV-Vis** (DMSO), λ_max_ (nm): 310, 430, 480.

## 7. Refinement

Crystal data, data collection and structure refinement details are summarized in Table 3 ▸. The hydrogen-atom positions were calculated geometrically at N—H = 0.88 Å and C—H = 0.95 Å and refined using a riding model with *U*_iso_(H) = 1.2 × *U*_eq_(N, C).

## Supplementary Material

Crystal structure: contains datablock(s) I. DOI: 10.1107/S2056989026001027/yy2020sup1.cif

Structure factors: contains datablock(s) I. DOI: 10.1107/S2056989026001027/yy2020Isup2.hkl

Supporting information file. DOI: 10.1107/S2056989026001027/yy2020Isup3.cml

CCDC reference: 2527280

Additional supporting information:  crystallographic information; 3D view; checkCIF report

## Figures and Tables

**Table 1:** Hydrogen-bond geometry (Å, °) *Cg*1 is the centroid of the C1–C6 ring.

*D*—H⋯*A*	*D*—H	H⋯*A*	*D*⋯*A*	*D*—H⋯*A*
N2—H2*A*⋯O1*B*^i^	0.88	2.07	2.939 (2)	168
N2—H2*B*⋯N3	0.88	2.31	2.672 (2)	104
C19—H19⋯*Cg*1^ii^	0.95	2.65	3.492 (2)	149

Symmetry codes: (i) 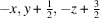; (ii) 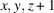.

**Table 2:** Selected interatomic distances (Å)

N2⋯O1*B*^i^	2.939 (2)	C6⋯H7	2.71
O1*A*⋯H6	2.44	C7⋯H20	2.66
O1*B*⋯H2	2.41	C7⋯H6	2.81
H2*A*⋯O1*B*^i^	2.07	C7⋯H10	2.58
N2⋯N3	2.672 (2)	C10⋯H7	2.75
N3⋯C20	2.913 (3)	H6⋯H7	2.39
N3⋯H2*B*	2.31	H7⋯H10	2.19
N3⋯H20	2.35		

Symmetry code: (i) 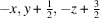.

**Table 3:** Experimental details

Crystal data	
Chemical formula	C_21_H_15_N_3_O_2_
*M*_r_	341.36
Crystal system, space group	Monoclinic, *P*2_1_/*c*
Temperature (K)	120
*a*, *b*, *c* (Å)	12.8213 (9), 15.7634 (9), 8.3763 (5)
β (°)	107.140 (7)
*V* (Å^3^)	1617.72 (18)
*Z*	4
Radiation type	Cu *K*α
μ (mm^−1^)	0.75
Crystal size (mm)	0.1 × 0.05 × 0.03

Data collection	
Diffractometer	XtaLAB Synergy R, DW system, HyPix
Absorption correction	Multi-scan (*CrysAlis PRO*; Rigaku OD, 2022 ▸)
*T*_min_, *T*_max_	0.419, 1.000
No. of measured, independent and observed [*I* > 2σ(*I*)] reflections	38890, 2880, 2609
*R*_int_	0.094
(sin θ/λ)_max_ (Å^−1^)	0.598

Refinement	
*R*[*F*^2^ > 2σ(*F*^2^)], *wR*(*F*^2^), *S*	0.053, 0.140, 1.07
No. of reflections	2880
No. of parameters	235
H-atom treatment	H-atom parameters constrained
Δρ_max_, Δρ_min_ (e Å^−3^)	0.25, −0.28

Computer programs: *CrysAlis PRO* (Rigaku OD, 2022 ▸), *SHELXT2018/2* (Sheldrick, 2015*a* ▸), *SHELXL2018/3* (Sheldrick, 2015*b* ▸) and *OLEX2* (Dolomanov *et al.*, 2009 ▸).

## supplementary crystallographic information

### (E)-2-[(Anthracen-9-ylmethylidene)amino]-4-nitroaniline Crystal data

**Table:**

C_21_H_15_N_3_O_2_	*F*(000) = 712
*M**_r_* = 341.36	*D*_x_ = 1.402 Mg m^−^^3^
Monoclinic, *P*2_1_/*c*	Cu *K*α radiation, λ = 1.54184 Å
*a* = 12.8213 (9) Å	Cell parameters from 19910 reflections
*b* = 15.7634 (9) Å	θ = 3.6–66.6°
*c* = 8.3763 (5) Å	µ = 0.75 mm^−^^1^
β = 107.140 (7)°	*T* = 120 K
*V* = 1617.72 (18) Å^3^	Prism, orange
*Z* = 4	0.1 × 0.05 × 0.03 mm

### (E)-2-[(Anthracen-9-ylmethylidene)amino]-4-nitroaniline Data collection

**Table:**

XtaLAB Synergy R, DW system, HyPix diffractometer	2880 independent reflections
Radiation source: Rotating-anode X-ray tube, Rigaku (Cu) X-ray Source	2609 reflections with *I* > 2σ(*I*)
Mirror monochromator	*R*_int_ = 0.094
Detector resolution: 10.0000 pixels mm^-1^	θ_max_ = 67.1°, θ_min_ = 3.6°
ω scans	*h* = −15→15
Absorption correction: multi-scan (CrysAlisPro; Rigaku OD, 2022)	*k* = −18→18
*T*_min_ = 0.419, *T*_max_ = 1.000	*l* = −9→9
38890 measured reflections	

### (E)-2-[(Anthracen-9-ylmethylidene)amino]-4-nitroaniline Refinement

**Table:**

Refinement on *F*^2^	Primary atom site location: dual
Least-squares matrix: full	Hydrogen site location: inferred from neighbouring sites
*R*[*F*^2^ > 2σ(*F*^2^)] = 0.053	H-atom parameters constrained
*wR*(*F*^2^) = 0.140	*w* = 1/[σ^2^(*F*_o_^2^) + (0.0631*P*)^2^ + 1.1761*P*] where *P* = (*F*_o_^2^ + 2*F*_c_^2^)/3
*S* = 1.07	(Δ/σ)_max_ < 0.001
2880 reflections	Δρ_max_ = 0.25 e Å^−^^3^
235 parameters	Δρ_min_ = −0.28 e Å^−^^3^
0 restraints	

### (E)-2-[(Anthracen-9-ylmethylidene)amino]-4-nitroaniline Special details

**Table:**

Geometry. All esds (except the esd in the dihedral angle between two l.s. planes) are estimated using the full covariance matrix. The cell esds are taken into account individually in the estimation of esds in distances, angles and torsion angles; correlations between esds in cell parameters are only used when they are defined by crystal symmetry. An approximate (isotropic) treatment of cell esds is used for estimating esds involving l.s. planes.

### (E)-2-[(Anthracen-9-ylmethylidene)amino]-4-nitroaniline Fractional atomic coordinates and isotropic or equivalent isotropic displacement parameters (Å^2^)

**Table:**

	*x*	*y*	*z*	*U*_iso_*/*U*_eq_	
O1A	0.11419 (12)	0.33297 (9)	0.7317 (2)	0.0445 (4)	
O1B	−0.01412 (13)	0.37615 (10)	0.8334 (2)	0.0488 (4)	
N3	0.25699 (13)	0.60375 (9)	0.5409 (2)	0.0288 (4)	
N1	0.05785 (14)	0.39013 (11)	0.7645 (2)	0.0360 (4)	
N2	0.12391 (14)	0.72245 (11)	0.5991 (3)	0.0419 (5)	
H2A	0.082433	0.763631	0.617169	0.050*	
H2B	0.176255	0.733782	0.554024	0.050*	
C5	0.17698 (14)	0.57686 (12)	0.6150 (2)	0.0264 (4)	
C21	0.40967 (15)	0.61781 (11)	0.3369 (3)	0.0285 (4)	
C8	0.43686 (15)	0.59916 (11)	0.5097 (2)	0.0277 (4)	
C16	0.49189 (16)	0.65214 (11)	0.2696 (3)	0.0308 (4)	
C7	0.35290 (15)	0.57129 (11)	0.5861 (2)	0.0280 (4)	
H7	0.370033	0.528682	0.670048	0.034*	
C4	0.10739 (15)	0.64265 (12)	0.6402 (2)	0.0300 (4)	
C6	0.16031 (15)	0.49425 (12)	0.6552 (2)	0.0286 (4)	
H6	0.205506	0.449991	0.636113	0.034*	
C14	0.62579 (15)	0.64459 (12)	0.5440 (3)	0.0316 (4)	
C15	0.59693 (16)	0.66442 (12)	0.3750 (3)	0.0333 (5)	
H15	0.650864	0.687170	0.330052	0.040*	
C3	0.02430 (16)	0.62217 (12)	0.7127 (3)	0.0318 (4)	
H3	−0.021416	0.665753	0.732859	0.038*	
C20	0.30485 (16)	0.60228 (12)	0.2218 (3)	0.0319 (4)	
H20	0.249363	0.577762	0.261119	0.038*	
C10	0.57722 (16)	0.59476 (12)	0.7901 (3)	0.0327 (4)	
H10	0.525540	0.572088	0.839747	0.039*	
C1	0.07603 (15)	0.47650 (12)	0.7242 (2)	0.0294 (4)	
C9	0.54482 (15)	0.61075 (11)	0.6148 (3)	0.0295 (4)	
C17	0.46355 (18)	0.67369 (12)	0.0969 (3)	0.0361 (5)	
H17	0.517281	0.698110	0.053462	0.043*	
C2	0.00881 (16)	0.53992 (13)	0.7543 (3)	0.0316 (4)	
H2	−0.047228	0.526443	0.803246	0.038*	
C19	0.28237 (17)	0.62179 (12)	0.0565 (3)	0.0345 (5)	
H19	0.212222	0.609453	−0.017515	0.041*	
C13	0.73284 (16)	0.66070 (13)	0.6513 (3)	0.0378 (5)	
H13	0.786633	0.682713	0.605152	0.045*	
C18	0.36215 (18)	0.66023 (13)	−0.0065 (3)	0.0372 (5)	
H18	0.344359	0.676395	−0.120670	0.045*	
C11	0.68046 (17)	0.61122 (13)	0.8878 (3)	0.0377 (5)	
H11	0.699748	0.599779	1.004218	0.045*	
C12	0.75971 (17)	0.64530 (14)	0.8178 (3)	0.0402 (5)	
H12	0.831298	0.657280	0.887402	0.048*	

### (E)-2-[(Anthracen-9-ylmethylidene)amino]-4-nitroaniline Atomic displacement parameters (Å^2^)

**Table:**

	*U*^11^	*U*^22^	*U*^33^	*U*^12^	*U*^13^	*U*^23^
O1A	0.0455 (9)	0.0245 (7)	0.0688 (11)	0.0010 (6)	0.0252 (8)	0.0059 (7)
O1B	0.0479 (9)	0.0385 (9)	0.0720 (11)	−0.0101 (7)	0.0364 (9)	0.0093 (8)
N3	0.0309 (8)	0.0226 (8)	0.0399 (9)	−0.0006 (6)	0.0214 (7)	−0.0013 (6)
N1	0.0343 (9)	0.0299 (9)	0.0462 (10)	−0.0051 (7)	0.0158 (8)	0.0043 (7)
N2	0.0407 (10)	0.0231 (9)	0.0747 (13)	0.0032 (7)	0.0369 (10)	0.0030 (8)
C5	0.0263 (9)	0.0250 (9)	0.0321 (10)	−0.0018 (7)	0.0150 (8)	−0.0003 (7)
C21	0.0336 (10)	0.0163 (8)	0.0427 (11)	0.0027 (7)	0.0223 (9)	−0.0031 (7)
C8	0.0306 (10)	0.0161 (8)	0.0426 (11)	0.0027 (7)	0.0206 (8)	−0.0024 (7)
C16	0.0375 (10)	0.0189 (9)	0.0441 (11)	0.0016 (7)	0.0244 (9)	−0.0042 (8)
C7	0.0325 (10)	0.0183 (8)	0.0381 (11)	−0.0009 (7)	0.0182 (8)	−0.0008 (7)
C4	0.0291 (10)	0.0245 (9)	0.0403 (11)	−0.0003 (7)	0.0165 (8)	−0.0010 (8)
C6	0.0284 (9)	0.0256 (10)	0.0348 (10)	−0.0002 (7)	0.0139 (8)	−0.0013 (8)
C14	0.0302 (10)	0.0225 (9)	0.0493 (12)	0.0022 (7)	0.0228 (9)	−0.0069 (8)
C15	0.0347 (11)	0.0256 (10)	0.0499 (12)	−0.0015 (8)	0.0285 (9)	−0.0063 (8)
C3	0.0278 (10)	0.0289 (10)	0.0443 (11)	0.0002 (7)	0.0194 (9)	−0.0037 (8)
C20	0.0345 (10)	0.0245 (9)	0.0433 (12)	0.0023 (8)	0.0218 (9)	−0.0025 (8)
C10	0.0336 (10)	0.0251 (10)	0.0446 (12)	0.0034 (8)	0.0197 (9)	−0.0022 (8)
C1	0.0298 (10)	0.0252 (9)	0.0359 (10)	−0.0039 (7)	0.0141 (8)	0.0025 (8)
C9	0.0306 (10)	0.0193 (9)	0.0448 (11)	0.0034 (7)	0.0207 (9)	−0.0045 (8)
C17	0.0460 (12)	0.0259 (10)	0.0467 (12)	−0.0027 (8)	0.0296 (10)	−0.0039 (8)
C2	0.0270 (9)	0.0345 (10)	0.0381 (11)	−0.0045 (8)	0.0167 (8)	−0.0012 (8)
C19	0.0376 (11)	0.0281 (10)	0.0417 (12)	0.0030 (8)	0.0177 (9)	−0.0050 (8)
C13	0.0296 (10)	0.0347 (11)	0.0555 (14)	−0.0008 (8)	0.0227 (10)	−0.0094 (9)
C18	0.0521 (13)	0.0282 (10)	0.0377 (11)	0.0017 (9)	0.0230 (10)	−0.0022 (8)
C11	0.0370 (11)	0.0350 (11)	0.0435 (12)	0.0060 (9)	0.0157 (9)	−0.0034 (9)
C12	0.0286 (10)	0.0395 (12)	0.0537 (14)	0.0009 (9)	0.0141 (9)	−0.0103 (10)

### (E)-2-[(Anthracen-9-ylmethylidene)amino]-4-nitroaniline Geometric parameters (Å, º)

**Table:**

O1A—N1	1.235 (2)	C14—C9	1.441 (3)
O1B—N1	1.244 (2)	C14—C13	1.424 (3)
N3—C5	1.412 (2)	C15—H15	0.9500
N3—C7	1.282 (2)	C3—H3	0.9500
N1—C1	1.438 (2)	C3—C2	1.372 (3)
N2—H2A	0.8800	C20—H20	0.9500
N2—H2B	0.8800	C20—C19	1.363 (3)
N2—C4	1.337 (3)	C10—H10	0.9500
C5—C4	1.424 (3)	C10—C9	1.425 (3)
C5—C6	1.377 (3)	C10—C11	1.361 (3)
C21—C8	1.417 (3)	C1—C2	1.391 (3)
C21—C16	1.439 (3)	C17—H17	0.9500
C21—C20	1.425 (3)	C17—C18	1.350 (3)
C8—C7	1.472 (2)	C2—H2	0.9500
C8—C9	1.416 (3)	C19—H19	0.9500
C16—C15	1.389 (3)	C19—C18	1.418 (3)
C16—C17	1.425 (3)	C13—H13	0.9500
C7—H7	0.9500	C13—C12	1.357 (3)
C4—C3	1.410 (3)	C18—H18	0.9500
C6—H6	0.9500	C11—H11	0.9500
C6—C1	1.396 (3)	C11—C12	1.420 (3)
C14—C15	1.389 (3)	C12—H12	0.9500
			
N2···O1B^i^	2.939 (2)	C6···H7	2.71
O1A···H6	2.44	C7···H20	2.66
O1B···H2	2.41	C7···H6	2.81
H2A···O1B^i^	2.07	C7···H10	2.58
N2···N3	2.672 (2)	C10···H7	2.75
N3···C20	2.913 (3)	H6···H7	2.39
N3···H2B	2.31	H7···H10	2.19
N3···H20	2.35		
			
C7—N3—C5	120.54 (16)	C2—C3—C4	120.67 (18)
O1A—N1—O1B	122.45 (17)	C2—C3—H3	119.7
O1A—N1—C1	119.49 (16)	C21—C20—H20	119.2
O1B—N1—C1	118.06 (17)	C19—C20—C21	121.54 (19)
H2A—N2—H2B	120.0	C19—C20—H20	119.2
C4—N2—H2A	120.0	C9—C10—H10	119.2
C4—N2—H2B	120.0	C11—C10—H10	119.2
N3—C5—C4	114.51 (16)	C11—C10—C9	121.50 (19)
C6—C5—N3	125.19 (16)	C6—C1—N1	119.07 (17)
C6—C5—C4	120.23 (16)	C2—C1—N1	119.07 (17)
C8—C21—C16	119.36 (18)	C2—C1—C6	121.86 (17)
C8—C21—C20	123.68 (17)	C8—C9—C14	118.87 (18)
C20—C21—C16	116.93 (18)	C8—C9—C10	123.50 (18)
C21—C8—C7	121.00 (17)	C10—C9—C14	117.55 (18)
C9—C8—C21	120.62 (17)	C16—C17—H17	119.2
C9—C8—C7	118.32 (18)	C18—C17—C16	121.55 (19)
C15—C16—C21	119.19 (19)	C18—C17—H17	119.2
C15—C16—C17	121.46 (18)	C3—C2—C1	119.28 (17)
C17—C16—C21	119.35 (19)	C3—C2—H2	120.4
N3—C7—C8	120.87 (17)	C1—C2—H2	120.4
N3—C7—H7	119.6	C20—C19—H19	119.5
C8—C7—H7	119.6	C20—C19—C18	121.0 (2)
N2—C4—C5	119.56 (17)	C18—C19—H19	119.5
N2—C4—C3	121.54 (17)	C14—C13—H13	119.3
C3—C4—C5	118.87 (17)	C12—C13—C14	121.47 (19)
C5—C6—H6	120.5	C12—C13—H13	119.3
C5—C6—C1	119.04 (17)	C17—C18—C19	119.5 (2)
C1—C6—H6	120.5	C17—C18—H18	120.2
C15—C14—C9	119.64 (18)	C19—C18—H18	120.2
C15—C14—C13	121.36 (18)	C10—C11—H11	119.6
C13—C14—C9	118.94 (19)	C10—C11—C12	120.8 (2)
C16—C15—H15	118.9	C12—C11—H11	119.6
C14—C15—C16	122.27 (18)	C13—C12—C11	119.8 (2)
C14—C15—H15	118.9	C13—C12—H12	120.1
C4—C3—H3	119.7	C11—C12—H12	120.1
			
O1A—N1—C1—C6	−2.7 (3)	C7—C8—C9—C14	−175.00 (15)
O1A—N1—C1—C2	176.97 (19)	C7—C8—C9—C10	1.7 (3)
O1B—N1—C1—C6	176.55 (18)	C4—C5—C6—C1	−1.5 (3)
O1B—N1—C1—C2	−3.8 (3)	C4—C3—C2—C1	−0.1 (3)
N3—C5—C4—N2	−2.2 (3)	C6—C5—C4—N2	−179.33 (19)
N3—C5—C4—C3	179.62 (17)	C6—C5—C4—C3	2.5 (3)
N3—C5—C6—C1	−178.32 (17)	C6—C1—C2—C3	1.1 (3)
N1—C1—C2—C3	−178.57 (18)	C14—C13—C12—C11	0.9 (3)
N2—C4—C3—C2	−179.8 (2)	C15—C16—C17—C18	178.65 (18)
C5—N3—C7—C8	−179.82 (16)	C15—C14—C9—C8	−0.5 (3)
C5—C4—C3—C2	−1.7 (3)	C15—C14—C9—C10	−177.41 (16)
C5—C6—C1—N1	179.36 (17)	C15—C14—C13—C12	176.69 (18)
C5—C6—C1—C2	−0.3 (3)	C20—C21—C8—C7	−7.4 (3)
C21—C8—C7—N3	−38.6 (3)	C20—C21—C8—C9	175.56 (16)
C21—C8—C9—C14	2.1 (3)	C20—C21—C16—C15	−176.78 (16)
C21—C8—C9—C10	178.78 (16)	C20—C21—C16—C17	4.0 (2)
C21—C16—C15—C14	0.1 (3)	C20—C19—C18—C17	3.5 (3)
C21—C16—C17—C18	−2.1 (3)	C10—C11—C12—C13	−0.8 (3)
C21—C20—C19—C18	−1.5 (3)	C9—C8—C7—N3	138.46 (18)
C8—C21—C16—C15	1.5 (3)	C9—C14—C15—C16	−0.6 (3)
C8—C21—C16—C17	−177.80 (16)	C9—C14—C13—C12	−0.3 (3)
C8—C21—C20—C19	179.61 (17)	C9—C10—C11—C12	0.2 (3)
C16—C21—C8—C7	174.45 (15)	C17—C16—C15—C14	179.34 (17)
C16—C21—C8—C9	−2.6 (3)	C13—C14—C15—C16	−177.57 (17)
C16—C21—C20—C19	−2.2 (3)	C13—C14—C9—C8	176.56 (16)
C16—C17—C18—C19	−1.6 (3)	C13—C14—C9—C10	−0.3 (3)
C7—N3—C5—C4	142.86 (18)	C11—C10—C9—C8	−176.31 (17)
C7—N3—C5—C6	−40.1 (3)	C11—C10—C9—C14	0.4 (3)

Symmetry code: (i) −*x*, *y*+1/2, −*z*+3/2.

### (E)-2-[(Anthracen-9-ylmethylidene)amino]-4-nitroaniline Hydrogen-bond geometry (Å, º)

*Cg*1 is the centroid of the C1–C6 ring.

**Table:**

*D*—H···*A*	*D*—H	H···*A*	*D*···*A*	*D*—H···*A*
N2—H2*A*···O1*B*^i^	0.88	2.07	2.939 (2)	168
N2—H2*B*···N3	0.88	2.31	2.672 (2)	104
C19—H19···*Cg*1^ii^	0.95	2.65	3.492 (2)	149

Symmetry codes: (i) −*x*, *y*+1/2, −*z*+3/2; (ii) *x*, *y*, *z*+1.
